# Beneficial Effects of 6-Month Supplementation with Omega-3 Acids on Selected Inflammatory Markers in Patients with Chronic Kidney Disease Stages 1–3

**DOI:** 10.1155/2017/1680985

**Published:** 2017-11-19

**Authors:** Agnieszka Pluta, Paweł Stróżecki, Jacek Kęsy, Kinga Lis, Beata Sulikowska, Grażyna Odrowąż-Sypniewska, Jacek Manitius

**Affiliations:** ^1^Division of Community Nursing, Faculty of Health Sciences, Ludwik Rydygier Collegium Medicum in Bydgoszcz, Nicolaus Copernicus University in Toruń, Łukasiewicza 1 Street, 85-821 Bydgoszcz, Poland; ^2^Department of Nephrology, Hypertension and Internal Diseases, Faculty of Medicine, Ludwik Rydygier Collegium Medicum in Bydgoszcz, Nicolaus Copernicus University in Toruń, M. Skłodowskiej-Curie 9 Street, 85-094 Bydgoszcz, Poland; ^3^Faculty of Biology and Environmental Protection, Nicolaus Copernicus University in Toruń, Lwowska 1 Street, 87-100 Toruń, Poland; ^4^Department of Allergology, Clinical Immunology and Internal Diseases, Faculty of Medicine, Ludwik Rydygier Collegium Medicum in Bydgoszcz, Nicolaus Copernicus University in Toruń, Ujejskiego 75 Street, 85-168 Bydgoszcz, Poland; ^5^Department of Laboratory Medicine, Faculty of Pharmacy, Ludwik Rydygier Collegium Medicum in Bydgoszcz, Nicolaus Copernicus University in Toruń, M. Skłodowskiej-Curie 9 Street, 85-094 Bydgoszcz, Poland

## Abstract

**Introduction:**

Chronic kidney disease (CKD) is accompanied by inflammation. The aim of this study was to evaluate the effect of 6-month supplementation with omega-3 acids on selected markers of inflammation in patients with CKD stages 1–3.

**Methods:**

Six-month supplementation with omega-3 acids (2 g/day) was administered to 87 CKD patients and to 27 healthy individuals. At baseline and after follow-up, blood was taken for C-reactive protein (CRP) and monocyte chemotactic protein-1 (MCP-1) concentration and white blood cell (WBC) count. Serum concentration of omega-3 acids—eicosapentaenoic acid (EPA), docosahexaenoic acid (DHA), and alpha-linolenic acid (ALA)—was determined using gas chromatography. And 24-hour urinary collection was performed to measure MCP-1 excretion.

**Results:**

After six-month omega-3 supplementation, ALA concentration increased in CKD patients and in the reference group, while EPA and DHA did not change. At follow-up, a significant decrease in urinary MCP-1 excretion in CKD (*p* = 0.0012) and in the reference group (*p* = 0.001) was found. CRP, serum MCP-1, and WBC did not change significantly. The estimated glomerular filtration rate (eGFR) did not change significantly in the CKD group.

**Conclusions:**

The reduction of urinary MCP-1 excretion in the absence of MCP-1 serum concentration may suggest a beneficial effect of omega-3 supplementation on tubular MCP-1 production.

**Trial Registration:**

This study was registered in ClinicalTrials.gov (identifier: NCT02147002).

## 1. Introduction

Chronic kidney disease (CKD) and its progression are closely linked to increased inflammatory response of the body. The severity of inflammation increases with the degree of renal failure [1, 2]. Markers of inflammation, such as high levels of C-reactive protein (CRP), interleukin-6 (IL-6), fibrinogen, and other acute phase proteins, such as lipoprotein(a), which are also atherogenic factors, are elevated in patients with CKD [2, 3]. On the other hand, reduced levels of proteins with antiatherogenic activity [e.g., apolipoprotein A-I and high-density lipoprotein (HDL)] are observed in CKD [3–5]. As a nonspecific marker of systemic inflammatory response, CRP activates the endothelium and accumulates in the atherosclerotic plaque, which suggests an important role in the local inflammatory process within a vessel wall [6, 7]. It was confirmed that reactions associated with inflammation developing in the vessel wall are responsible for the creation and development of atherosclerotic lesions in the population of patients with CKD [8]. As regards inflammatory response in the kidneys, monocyte chemotactic protein-1 (MCP-1), belonging to the group of C-C chemokines, cannot be overlooked. This protein is produced by many cells, particularly tubular epithelium and inflowing monocytes and macrophages [9]. In the epithelial cells of renal tubules, MCP-1 stimulates the transcription of nuclear factor-kappa B (NF-kB) and activator protein-1 (AP-1), that is, transcription factors leading to increased production of interleukin-6 (IL-6) [10]. The most important task of IL-6 is to participate in the immune and inflammatory response [11]. Through extracellular signal-regulated kinase, MCP-1 induces the proliferation of vascular smooth muscle cells, contributing to the progression of renal damage [12].

Among polyunsaturated fatty acids, omega-3 or n-3 (*α*-linolenic acid, 18:3, ALA) and omega-6 or n-6 (linoleic acid, 18:2, LA) are of particular importance [13]. Several recent studies have demonstrated the beneficial effects of polyunsaturated fatty acids in reducing the inflammation and the development of atherosclerosis in the population of patients with CKD [14, 15]. However, a different study did not confirm such a relationship [16].

The aim of this study was to evaluate the effect of 6-month supplementation with omega-3 acids on selected inflammatory markers in patients with CKD stages 1–3.

## 2. Methods

The study was conducted between September 2012 and November 2014 upon approval by the Bioethics Committee of the Nicolaus Copernicus University in Toruń Ludwik Rydygier Collegium Medicum in Bydgoszcz [KB 305/2012]. All patients included in the study provided written consent for participation.

The study population consisted of patients with CKD receiving treatment in the Nephrology Outpatient Clinic of University Hospital No. 1 in Bydgoszcz. The criteria for inclusion in the study were as follows: diagnosed chronic kidney disease stages 1–3, written consent from the patient to participate in the study, and age over 18 years.

Exclusion criteria included immunosuppressive therapy, diabetes, and lack of consent to participate in the study. 130 patients were asked to participate in the study; 40 refused to participate. Thirty patients without CKD, hypertension, or overt cardiovascular disease were classified as the reference group.

In the analyzed population of CKD patients, 30 patients were in stage 1, 33 in stage 2, and 27 in stage 3 of the disease.

During the 6-month supplementation, one woman from the experimental group and one from the reference group were excluded, as they became pregnant. Two patients with CKD and two patients from the reference group failed to attend the follow-up examination after the 6-month supplementation with omega-3 acids. For the purposes of this study, the results of 87 patients with CKD and 27 patients from the reference group were analyzed both before and after supplementation.

The underlying kidney diseases in the CKD group were as follows: chronic glomerulonephritis confirmed by kidney biopsy (*n* = 16; 18.4%), hypertensive nephrosclerosis (*n* = 3; 3.5%), polycystic kidney disease (*n* = 28; 32.2%), gouty nephropathy (*n* = 5; 5.7%), nephrolithiasis (*n* = 23; 26.4%), and loss of one kidney due to injury (*n* = 1, 1.1%). In 11 cases (12.6%), the cause of the disease was unknown.

Each patient participating in the study received 6-month supplementation with omega-3 (Gold Omega 3) at a dose of 2 × 1000 mg. One capsule of Gold Omega 3 = 1000 mg contains 65% omega-3 acid, including 330 mg of eicosapentaenoic acid (EPA), 220 mg of docosahexaenoic acid (DHA), and 100 mg of other acids including alpha-linolenic acid (ALA).

All persons participating in the study had their body mass index (BMI) calculated. Measurements were also taken of their waist and hip circumference.

Fasting blood was collected for the determination of serum creatinine, CRP, and the concentration of MCP-1. Peripheral blood leukocyte count was performed (WBC). In the 24-hour urine collection, the excretion of MCP-1 and excretion of creatinine were determined. The rate of MCP-1 excretion with urine was expressed in ng per 1 mg of creatinine in urine.

### 2.1. Laboratory Tests

Material used for the study was venous blood serum. Fasting blood was collected from the median cubital vein into two dry glass tubes without additives, in the Vacutainer closed vacuum system under standard conditions, between 7:00 and 9:00 in the morning. After collection, blood samples were left at room temperature for 30 minutes for clotting. One tube was used for the enzymatic creatinine assay using the Horiba ABX Pentra 400 biochemical analyzer. The estimated glomerular filtration rate (eGFR) was then estimated on the basis of CKD-EPI [17]. The second tube of coagulated blood was centrifuged for 15 minutes at 4000 RPM. After centrifugation, serum was separated from the blood clot. The separated serum at a volume of about 2 ml was stored in an Eppendorf tube at −80°C. For the analysis of ALA, EPA, and DHA, a PerkinElmer (USA) gas chromatograph equipped with a flame ionization detector (GC-FID) was used. Separation of FAMEs was carried out on an Equity-5 (Supelco) capillary column (30 m × 0.25 mm i.d., 0.25 *μ*m film thickness) using hydrogen as the carrier gas.

100 *μ*l of human serum was saponified in 5 ml PTFE screw-capped glass tubes containing 10 *μ*g of tridecanoic acid as an internal standard and 1 ml of 0.5% (w/v) sodium methylate. The samples were heated for 15 min at a temperature of 100°C and, after cooling to room temperature, esterified with 1.5 ml of BF_3_ in methanol (also at 100°C) for 10 min. Again after cooling of the tubes, 1 ml of *n*-hexane was added to extract the fatty acid methyl esters. The contents of the tubes were then shaken for 1 min and 1 ml of saturated sodium chloride solution was added. Afterwards, the tubes were centrifuged for 5 min at 2200 ×g. The clear hexane top layer was transferred into an injection vial, evaporated to dryness under a stream of nitrogen, and then redissolved in 100 *μ*l of hexane. 1 *μ*l of the final solution was applied into the GC injector. The method of FA analysis was adopted from Bondia-Pons et al. [18].

The identities of sample methyl ester peaks were determined by comparing their relative retention times with those of well-known FAME standards. Quantification was based on the amount of the internal standard recovered. The results were expressed in mg/100 ml of serum:

(1) $$\begin{array}{c} Concentration\;\;of\;\;KT=\frac{S\;\;KT}{S\;\;ST}\times M\;\;ST\;\left[\;mg/100\;ml\;\right], \end{array}$$

where S KT is the fatty acid peak area, S ST is the internal standard peak area, and M ST is the amount of internal standard in *μ*g.

Serum levels of creatinine and C-reactive protein were determined using the Horiba ABX Pentra 400 biochemical analyzer. The number of leukocytes was determined using a conventional method.

### 2.2. Statistical Analysis

Distribution of variables was tested using the Shapiro-Wilk test. For comparison of results in more than two groups, the nonparametric ANOVA test was used. As a post hoc test for the detailed identification of statistically different groups, the Tukey test was used. Data were presented as mean ± standard deviation (SD), and the median and top and bottom quartiles were given for variables that were not normally distributed. Evaluation of the correlations between the analyzed indicators was carried out using Pearson's correlation coefficient (for samples with normal distribution) and Spearman's correlation coefficient (for samples with nonnormal distribution). The level of statistical significance was set at *p* < 0.05. Changes (Δ) in selected parameters were calculated. Δ was calculated by subtracting the initial value from the final value.

## 3. Results

Clinical characteristics and laboratory results in patients with CKD stages 1–3 and in the reference group before and after 6 months of supplementation with omega-3 acid are shown in Table 1. The mean age of CKD patients was 57 ± 11, and in the reference group it was 54 ± 11. In patients with CKD and in the reference group, there was a statistically significant increase in the concentration of ALA after supplementation. Creatinine concentration and eGFR did not change in patients with CKD, while in the reference group, eGFR decreased and serum creatinine concentration increased. The clinical characteristics and laboratory results in the different stages of CKD are shown in Table 2.

During the supplementation with omega-3 acid, the tolerance of the Gold Omega 3 preparation was good. The occurrence of side effects in the form of belching and nausea was observed in 2 patients (2.2%). The symptoms were transient and did not require the discontinuation of therapy. In the group of patients with CKD, a statistically significant negative correlation between eGFR and CRP (*R* = −0.27; *p* = 0.009) was demonstrated prior to supplementation. No statistically significant correlation was found between the concentrations of EPA, DHA, and ALA and markers of inflammation. In the reference group, a statistically significant negative correlation between the concentration of ALA and the number of WBC (*R* = −0.46; *p* = 0.014) was demonstrated before supplementation. In patients with CKD, a positive correlation between serum levels of MCP-1 and CRP (*R* = 0.38; *p* = 0.0002) and a negative correlation between serum levels of MCP-1 and eGFR (*r* = −0.29; *p* = 0.006) were found after supplementation.

In the entire group of patients with CKD stages 1–3, no statistically significant correlations were shown between Δ of EPA, DHA, and ALA concentration and Δ of inflammatory markers. In patients with stage 2 CKD, a statistically significant negative correlation was found between Δ of ALA concentration and Δ of 24-hour urinary excretion of MCP-1 (*r* = −0.37; *p* < 0.05).

## 4. Discussion

Irrespective of its cause, chronic kidney disease is characterized by accelerated atherosclerosis and vascular stiffening with ventricular remodeling, which increases the risk of cardiovascular events [19, 20]. It is accompanied by inflammation, regardless of its causes, stage of disease, or age of the patient.

Recent investigations have provided more and more information on the favorable effects of omega-3 enriched diet and omega-3 supplementation on the inflammatory process, reducing the risk of cardiovascular events in the population of patients with CKD and slowing the progression of CKD [21–23]. Omega-3 fatty acids are the precursors of the synthesis of inflammatory mediators. EPA and DHA, which replace arachidonic acid in plasma membranes, reduce the amount of the substrate, out of which proinflammatory eicosanoids are formed under the influence of cyclooxygenase-2 (COX-2) and lipoxygenases (LOX). Omega-3 fatty acids reduce the production of prostaglandins and 2-series thromboxane, 4-series leukotrienes, and 5-hydroxyeicosatetraenoic acid (5-HETE). An alternative substrate for COX-2 and LOX is EPA, which produces prostaglandins and thromboxane (TxA3) and consequently leads to the formation of TxA2 and 5-series leukotrienes, compounds with much weaker proinflammatory properties [22]. TxA2 enhances, among other things, the proliferation of mesangial cells in the kidney [24]. EPA and DHA are precursors of molecules responsible for the suppression of the inflammatory process. The said molecules include E-series resolvins, synthesized from EPA by LOX, as well as D-series resolvins, D-series protectins, and maresins derived from DHA [25].

The main finding of the present study was that the urinary excretion of MCP-1 in the urine decreased after 6-month supplementation with omega-3 acid in both CKD and the reference group, with no change in the serum concentration of MCP-1. The largest decrease in MCP-1 in the urine was observed in patients with stage 2 CKD (*p* < 0.001). This could indicate that omega-3 acids inhibit the production of MCP-1 by tubular epithelial cells. The beneficial effect of supplementation with omega-3 on reducing the concentration of MCP-1 in serum has so far been revealed in only one study, which involved patients with stage 5 CKD undergoing hemodialysis and receiving EPA + DHA at a dose of 2.9 g/day for 12 weeks. Urinary excretion of MCP-1 was not studied due to the stage of disease [26]. Experimental studies conducted on male Sprague-Dawley rats with CKD receiving omega-3 acid for 12 weeks revealed that there was a decrease in MCP-1 in serum [27]. One of the conclusions drawn by the authors was that long-term supplementation with omega-3 acids attenuated tubulointerstitial fibrosis in the remnant kidney.

Inflammation of the renal interstitium increases the progression of CKD. A significant role in this respect is played by proinflammatory chemokine MCP-1, which induces the activation and migration of monocytes/macrophages, basophils, CD4 lymphocytes, and NK cells [28]. In the kidneys, MCP-1 is synthesized via the activation of the NF-*κ*B in renal tubular epithelial cells and mesangial cells [9, 29]. Through extracellular signal-regulated kinase, MCP-1 induces the proliferation of vascular smooth muscle cells, contributing to the progression of kidney damage [10, 29, 30]. Therefore, reduced production of MCP-1 in the renal interstitium may contribute to slowing the progression of CKD by inhibiting smooth muscle proliferation. This hypothesis, however, would have to be verified by experimental studies.

The use of omega-3 acids contributes to the downward trend of daily MCP-1 urinary excretion. The largest decrease was observed in patients with CKD stage 2, who constituted the largest study group. It cannot be ruled out that if the remaining groups were bigger, a decrease in daily MCP-1 urinary excretion could be observed. In stage 2 CKD, the decrease in daily MCP-1 urinary excretion was the greatest and amounted to 42%, while in CKD 3 the decrease was 28%. This suggests that the beneficial effect of omega-3 on the kidney interstitium is more favorable in the early stages of the disease due to a less advanced process of kidney interstitial fibrosis.

The present study did not reveal any effects of supplementation with omega-3 fatty acids on serum CRP levels. Similarly, no beneficial effects of fatty acids on the levels of serum CRP and IL-6 were shown in patients undergoing peritoneal dialysis and hemodialysis who received 8-week supplementation with omega-3 acids at a dose of 3 g/day [28, 29, 31, 32]. In a study of CKD patients with serum creatinine in the range of 150 to 400 micromoles/l after receiving 2-month supplementation with omega-3 acid at a dose of 2.4 g/day, the authors found only a tendency to reduce the concentration of hsCRP [33]. Furthermore, Deike et al., who studied a group of patients with CKD not treated with dialysis and receiving 8-week supplementation with omega-3 acid at a dose of 1.4 g EPA + DHA 1 g/day, confirmed the lack of anti-inflammatory effects of omega-3 acid [16].

## 5. Conclusions

The reduction of urinary MCP-1 excretion in the absence of MCP-1 serum concentration may suggest a beneficial effect of omega-3 supplementation on tubular MCP-1 production. Our results suggest a potential favorable effect of omega-3 supplementation on renal interstitial inflammation.

## Figures and Tables

**Table 1:** Characteristics of the studied group of patients with CKD and the reference group at baseline and after supplementation with omega-3 acid.

Parameter	Reference (*n* = 27)		*p* (1) vs (2)	CKD (*n* = 87)		*p* (3) versus (4)
	Baseline (1)	Postintervention (2)		Baseline (3)	Postintervention (4)	
Gender						
Women/men	18/9	18/9		40/47	40/47	
BMI (kg/m^2^)	24.5 ± 2.9	24.7 ± 2.8	0.053	27.2 ± 3.9	27.2 ± 3.8	0.78
Waist circumference (cm)	88.4 ± 11.4	84.7 ± 11.4	0.92	94.6 ± 12.6	94.4 ± 12.4	0.67
Hip circumference (cm)	101.1 ± 5.6	101.2 ± 5.2	0.94	105.7 ± 7.9	104.7 ± 8.3	0.016
Creatinine (mg/dl)	0.75 ± 0.20	0.82 ± 0.21	0.0097	1.05 ± 0.35	1.12 ± 0.46	0.30
eGFR CKD-EPI (ml/min/1.73 m^2^)	96.3 ± 14.8	89.9 ± 14.9	0.011	74.9 ± 23.5	72.3 ± 25.5	0.055
ALA (mg/100 ml)	1.52 (1.17; 2.11)	2.48 (2.06; 3.38)	0.0008	1.8 (1.11; 2.64)	3.0 (2.24; 3.96)	0.0001
EPA (mg/100 ml)	6.64 ± 3.42	7.25 ± 3.51	0.52	7.6 ± 3.83	8.46 ± 4.95	0.20
DHA (mg/100 ml)	8.21 ± 4.67	8.31 ± 3.36	0.92	9.09 ± 4.48	8.95 ± 3.84	0.81
CRP (mg/l)	0.42 (0.15; 1.99)	0.48 (0.16; 1.30)	0.79	1.03 (0.25; 2.73)	1.17 (0.40; 2.99)	0.23
WBC (10^3^/*µ*l)	5.9 ± 1.8	5.5 ± 1.6	0.06	6.4 ± 1.7	6.3 ± 1.5	0.51
MCP-1 serum (pg/dl)	328.4 ± 112.4	374.4 ± 116.9	0.12	343.2 ± 147	353.1 ± 123.8	0.45
MCP-1 urinary excretion (ng/24 h)	324.8 (235.7; 418.7)	208.7 (99.9; 311.5)	0.001	427.1 (284.8; 645.1)	299.7 (155.9; 467.3)	0.0012
MCP-1 urinary excretion (ng)/creatinine urinary excretion (mg)	0.411 (0.276; 0.775)	0.186 (0.106; 0.322)	0.00006	0.514 (0.315; 0.817)	0.338 (0.201; 0.606)	0.00098

ALA: alpha-linolenic acid; BMI: body mass index; CRP: C-reactive protein; DHA: docosahexaenoic acid; eGFR: estimated glomerular filtration rate; EPA: eicosapentaenoic acid; MCP-1 serum: monocyte chemotactic protein 1 in serum; MCP-1 urinary excretion: 24-hour urinary excretion of monocyte chemotactic protein 1; WBC: white blood cells. MCP-1 urinary excretion/creatinine urinary excretion of creatinine: monocyte chemotactic protein 1 excretion with urine was expressed in ng per 1 mg of creatinine in urine.

**Table 2:** Characteristics of the studied group of patients with CKD 1, CKD 2, and CKD 3 at baseline and after supplementation with omega-3 acid.

Parameter	CKD 1 (*n* = 29)	CKD 2 (*n* = 32)	CKD 3 (*n* = 26)	ANOVA *p*
Gender				
Women/men	14/15	15/17	11/15	
Age (years)				
Baseline	50 ± 11	58 ± 11^1^	63 ± 7^2^	0.000035
Postintervention	51 ± 11	59 ± 11^1^	64 ± 7^2^	0.000035
BMI (kg/m^2^)				
Baseline	26.4 ± 4.0	27.6 ± 3.9	27.6 ± 3.7	0.44
Postintervention	26.7 ± 3.9	27.3 ± 3.7	27.6 ± 4.0	0.67
Waist circumference (cm)				
Baseline	91.3 ± 13.6	94.6 ± 12.5	98.2 ± 11.1	0.13
Postintervention	92.0 ± 12.6	93.4 ± 11.8	98.1 ± 12.5	0.17
Hip circumference (cm)				
Baseline	104.4 ± 8.6	106.4 ± 7.4	106.3 ± 8.0	0.58
Postintervention	103.7 ± 8.2	105.3 ± 7.6^*∗*^	105.3 ± 9.4	0.70
Creatinine (mg/dl)				
Baseline	0.75 ± 0.17	1.00 ± 0.16^2^	1.46 ± 0.29^1,3^	0.001
Postintervention	0.78 ± 0.16	1.04 ± 0.24^1^	1.58 ± 0.51^1,4^	0.0001
eGFR CKD-EPI (ml/min/1.73 m^2^)				
Baseline	101.1 ± 9.5	74.2 ± 9.0^2^	46.6 ± 8.1^2,4^	0.001
Postintervention	97.3 ± 12.3	72.±17.2^2^	44.7 ± 13.4^2,4^	0.001
ALA (mg/100 ml)				
Baseline	1.36 (0.96; 2.47)	1.98 (1.48; 2.57)	1.65 (1.11; 2.77)	0.33
Postintervention	3.03 (2.07; 3.83)^*∗∗*^	3.22 (2.31; 4.79)^*∗∗*^	2.82 (2.24; 3.56)^*∗*^	0.55
EPA (mg/100 ml)				
Baseline	7.51 ± 3.87	8.25 ± 4.45	6.90 ± 2.85	0.42
Postintervention	8.45 ± 4.22	7.81 ± 4.21	9.26 ± 6.42	0.54
DHA (mg/100 ml)				
Baseline	8.77 ± 5.19	9.37 ± 4.25	9.11 ± 4.02	0.88
Postintervention	8.67 ± 2.799	8.14 ± 3.63	10.25 ± 4.80	0.10
CRP (mg/l)				
Baseline	0.28 (0.10; 1.06)	1.67 (0.51; 3.17)^1^	1.17 (0.47; 2.40)^5^	0.0055
Postintervention	0.77 (0.35; 2.82)^*∗*^	2.13 (0.61; 3.22)	0.99 (0.46; 2.30)	0.23
WBC (10^3^/*µ*l)				
Baseline	6.5 ± 1.9	6.0 ± 1.4	6.7 ± 1.7	0.33
Postintervention	6.2 ± 1.7	6.2 ± 1.5	6.7 ± 1.4	0.37
MCP-1 serum (pg/dl)				
Baseline	316.5 ± 108.4	370.5 ± 158.3	340.5 ± 169.6	0.37
Postintervention	315.3 ± 88.3	359.1 ± 108.6	388.0 ± 162.0^*∗*^	0.08
MCP-1 urinary excretion (ng/24 h)				
Baseline	362.6 (275.8; 695.2)	432.8 (263.2; 614.1)	458.4 (311.6; 697.8)	0.50
Postintervention	354.63 (114.8; 481.5)	251.1 (115.3; 437.8)^*∗∗*^	329.5 (259.2; 468.8)	0.22

ALA: alpha-linolenic acid; BMI: body mass index; CRP: C-reactive protein; DHA: docosahexaenoic acid; eGFR: estimated glomerular filtration rate; EPA: eicosapentaenoic acid; MCP-1 serum: monocyte chemotactic protein 1 in serum; MCP-1 urinary excretion: 24-hour urinary excretion of monocyte chemotactic protein 1; WBC: white blood cells. ^*∗*^*p* < 0.05 after versus before; ^*∗∗*^*p* < 0.001 after versus before; ^1^*p* < 0.01 versus CKD 1; ^2^*p* < 0.001 versus CKD 1; ^3^*p* < 0.01 versus CKD 2; ^4^*p* < 0.001 versus CKD 2; ^5^*p* < 0.05 versus CKD 1.

## Additional Points

*Critical Comments about the Materials and Methods*. The present study has several limitations. These limitations result from a relatively small study group and a short period of supplementation with omega-3 or the daily dose of omega-3 itself. Extending the observation period to up to 12 months will improve the chances of demonstrating the effect of omega-3 on the inflammatory process. The study did not include patients with CKD who did not receive supplementation with omega-3 acids and who would serve as a reference group.
